# An Uncommon Cause of Angina

**DOI:** 10.3390/diagnostics15172241

**Published:** 2025-09-04

**Authors:** David S. Majdalany, Elaina A. Blickenstaff, Francois Marcotte, Jason H. Anderson

**Affiliations:** 1 Department of Cardiovascular Medicine, Mayo Clinic, 200 First Street SW, Rochester, MN 55905, USA; 2 Joan C. Edwards School of Medicine, Marshall University, One John Marshall Drive, Huntington, WV 25755, USA; 3 Mayo Clinic, 13400 E Shea Boulevard, Scottsdale, AZ 85259, USA

**Keywords:** anomalous right coronary artery from the pulmonary artery (ARCAPA), angina, chest pain, computed tomography, coronary angiography

## Abstract

Coronary anomalies, although rare, should be considered when young patients present with angina. Clinical suspicion and multi-modality imaging including coronary angiography and tomographic imaging should be pursued for symptomatic patients such as the one we are presenting with anomalous right coronary artery from the pulmonary artery. She was promptly referred for surgical intervention with re-implantation of the right coronary artery onto the aorta.

**Figure 1:**
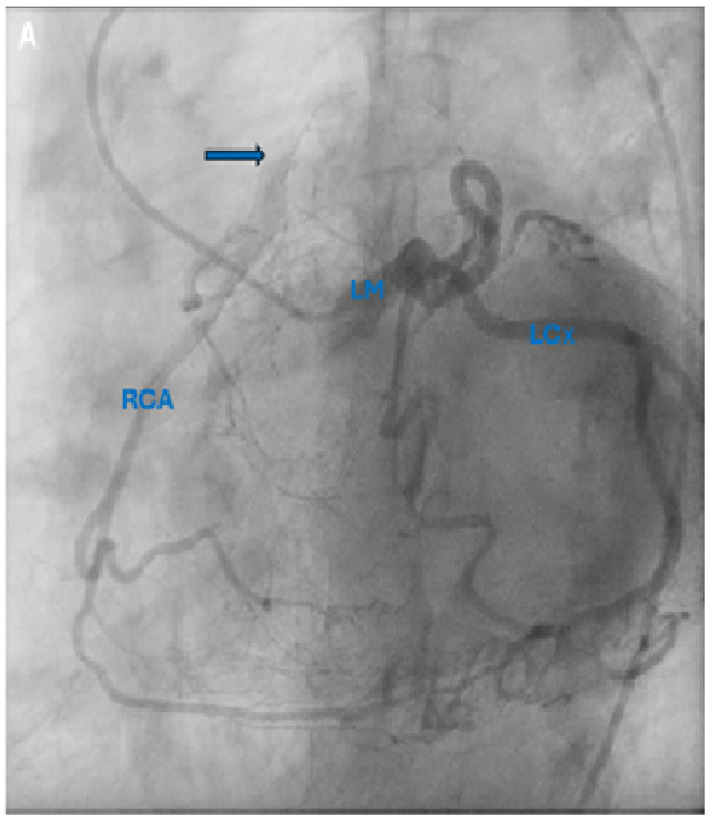

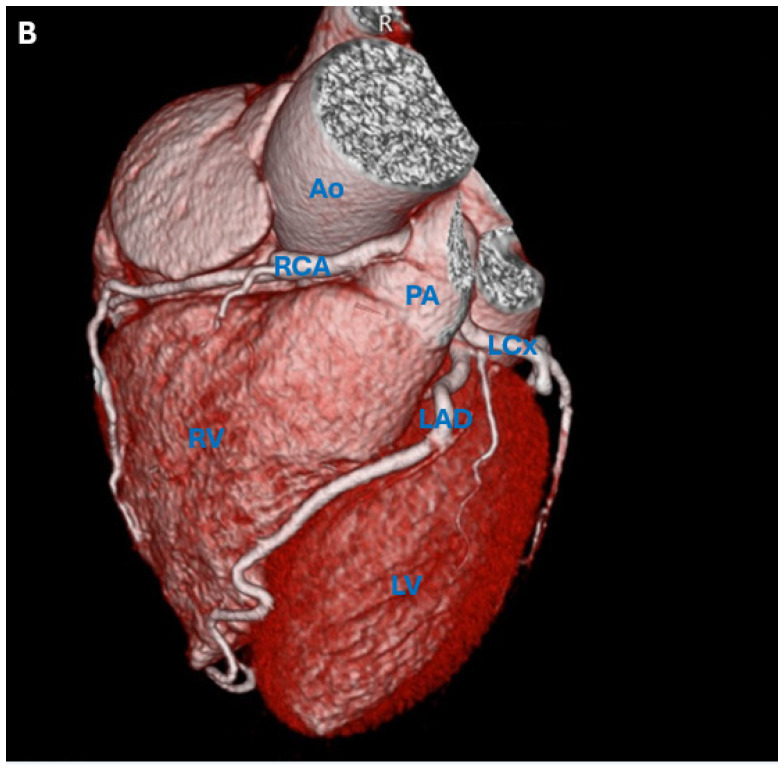
(**A**). Coronary angiogram revealing opacification of a hypoplastic right coronary artery (RCA) via collaterals with contrast injection in the dilated left coronary artery and retrograde flow into the pulmonary artery (arrow) representing a mild degree of coronary steal. (**B**). Computerized tomography coronary angiogram demonstrating anomalous patent right coronary artery arising from the pulmonary artery. Ao: aorta; LAD: left anterior descending coronary artery; LCx: left circumflex coronary artery; LM: left main coronary artery; LV: left ventricle; RCA: right coronary artery; RV: right ventricle. A 52-year-old female with past medical history of hypertension and hyperlipidemia experienced six months of worsening exertional chest tightness radiating to her throat. Given concerning symptoms for angina, she underwent coronary angiography (panel A, Video S1) which revealed opacification of a hypoplastic right coronary artery (RCA) via collaterals with contrast injection in the dilated left coronary artery and retrograde flow into the pulmonary artery (arrow) representing a mild degree of coronary steal. Her computerized tomography coronary angiogram (panel B, Video S2) confirmed anomalous patent right coronary artery arising from the pulmonary artery 10 mm cranial to the pulmonic valve with a widely patent orifice and evidence of retrograde flow from the right coronary artery into the pulmonary artery. On echocardiography, the patient had normal biventricular size and systolic function with no significant valvular abnormalities. Given the worsening exertional symptoms, she was referred for surgical intervention and underwent re-implantation of the RCA onto the aorta, which was the most common treatment strategy (69%) reported in a systematic review of 223 cases of ARCAPA [1]. Her post-operative course was unremarkable with widely patent RCA–aorta anastomosis on tomographic imaging. She was dismissed home four days after her surgery with echocardiography revealing normal biventricular size and function. Anomalous origin of the right coronary artery from the pulmonary artery is a rare congenital cardiac lesion found in <0.002% of the population, with its first description in 1885 [2,3]. It can lead to myocardial ischemia, heart failure, and sudden cardiac arrest. Symptomatic patients with ARCAPA have a bimodal age distribution at presentation, the first near birth and the second around 50 years when it is noted incidentally [1]. This is in contrast to patients with anomalous origin of the left coronary artery from the pulmonary artery that typically present in infancy (85%) with congestive heart failure due to myocardial ischemia resulting in left ventricular dysfunction and mitral regurgitation with 90% mortality if left untreated [4,5]. Surgical intervention is a class I indication for symptomatic patients attributable to ARCAPA [6,7]. Multi-modality imaging is important in the diagnosis, with coronary angiography being the most common mode of diagnosis (40%) reported in a systematic review of 223 cases of ARCAPA [1,8]. In our case, coronary angiography delineated the coronary anomaly and showcased the retrograde flow from the coronary arteries to the pulmonary artery. This was further confirmed on computed tomography, which provided a three-dimensional representation of cardiac anatomy, with echocardiography revealing normal biventricular function and no wall motion or valvular abnormalities. Moreover, surgical intervention can include either re-implantation of the RCA onto the aorta or ligation of the RCA with coronary bypass grafting to provide dual coronary circulation [6]. The latter approach has the drawback of decreased patency rate compared to re-implantation. Mortality post operative intervention was low at 2.5% (4 deaths in 157 surgical cases), with good early and mid-term results, albeit there are limited reports of follow-up post-surgical correction for ARCAPA [1,9,10]. In a report of seven cases of ARCAPA that underwent repair, one patient died post-RCA ligation on post-operative day 1 and one patient developed a thrombus in the dilated RCA which resolved after anticoagulation. In a cohort of 70 published cases of ARCAPA, there were 13 reported deaths, including 4 cases of sudden death and 4 perioperative deaths [8].

## Data Availability

No new data were created or analyzed in this study. Data sharing is not applicable to this article.

## Supplementary Materials

The following supporting information can be downloaded at: https://www.mdpi.com/article/10.3390/diagnostics15172241/s1, Video S1: Coronary angiogram revealing ARCAPA; Video S2: Cardiac computed Tomography revealing ARCAPA.
